# On the Solutions of Some Linear Complex Quaternionic Equations

**DOI:** 10.1155/2014/563181

**Published:** 2014-07-02

**Authors:** Cennet Bolat, Ahmet İpek

**Affiliations:** ^1^Department of Mathematics, Faculty of Art and Science, Mustafa Kemal University, Tayfur Sökmen Campus, 31100 Hatay, Turkey; ^2^Department of Mathematics, Faculty of Kamil Özdağ Science, Karamanoğlu Mehmetbey University, 70100 Karaman, Turkey

## Abstract

Some complex quaternionic equations in the type *AX* − *XB* = *C* are investigated. For convenience, these equations were called generalized Sylvester-quaternion equations, which include the Sylvester equation as special cases. By the real matrix representations of complex quaternions, the necessary and sufficient conditions for the solvability and the general expressions of the solutions are obtained.

## 1. Introduction

Mathematics, as with most subjects in science and engineering, has a long and varied history. In this connection one highly significant development which occurred during the nineteenth century was the quaternions, which are the elements of noncommutative algebra. Quaternions have many important applications in many applied fields, such as computer science, quantum physics, statistic, signal, and color image processing, in rigid mechanics, quantum mechanics, control theory, and field theory; see, for example, [1].

In recent years, quaternionic equations have been investigated by many authors. For example, the author of the paper [2] classified solutions of the quaternionic equation *ax* + *xb* = *c*. In [3], the linear equations of the forms *ax* = *xb* and $ax=\bar{x}b$ in the real Cayley-Dickson algebras (quaternions, octonions, and sedenions) are solved and the form for the roots of such equations is established. In [4], the solutions of the equations of the forms *ax* = *xb* and $ax=\bar{x}b$ for some generalizations of quaternions and octonions are investigated. In [5], the *αxβ* + *γxδ* = *ρ* linear quaternionic equation with one unknown, *αxβ* + *γxδ* = *ρ*, is solved. In [6], Bolat and İpek first defined the quaternion intervals set and the quaternion interval numbers, second, they presented the vector and matrix representations for quaternion interval numbers and then investigated some algebraic properties of these representations, and finally they computed the determinant, norm, inverse, trace, eigenvalues, and eigenvectors of the matrix representation established for a quaternion interval number. In [7], the quaternionic equation *ax* + *xb* = *c* is studied. In [8], Bolat and İpek first considered the linear octonionic equation with one unknown of the form *α*(*xα*) = (*αx*)*α* = *α* *xα* = *ρ*, with 0 ≠ *α* ∈ **O**; second, they presented a method which allows to reduce any octonionic equation with the left and right coefficients to a real system of eight equations and finally reached the solutions of this linear octonionic equation from this real system. In [9], Flaut and Shpakivskyi investigated the left and right real matrix representations for the complex quaternions. The theory of the quaternion equations and matrix representations of quaternions is considered completely in [2–14].

In this paper, we aim to obtain the solutions of some linear equations with two terms and one unknown by the method of matrix representations of complex quaternions over the complex quaternion field and to investigate the solutions of some complex quaternionic linear equations.

The paper is organized as follows. In Section 2, we start with some basic concepts and results from the theory of the quaternion equations and matrix representations of quaternions which are necessary for the following. In Section 3, we obtain the solutions of some linear equations with two terms and one unknown by the method of matrix representations of complex quaternions over the complex quaternion field. We finish the paper with some conclusions about the study presented.

## 2. Preliminaries

The following notations, definitions, propositions, lemmas, and theorems will be used to develop the proposed work. We now start the definitions of the quaternion and complex quaternion and their basic properties that will be used in the sequel.

It is well known that a complex number is a number consisting of a real and imaginary part. It can be written in the form *a* + *bi*, where *i* is the imaginary unit with the defining property *i* ^2^ = −1. The set of all complex numbers is usually denoted by *C*. From here, it can be easily said that the set of complex numbers is an extension of the set of real numbers, usually denoted by *R*. That is, *R* ⊂ *C*.

In the literature, firstly, the set of quaternions introduced as

(1) $$\begin{array}{c} H_{R}=\left\{a=a_{0}+a_{1}i+a_{2}j+a_{3}k:a_{s}\in R,s=0,1,2,3\right\}, \end{array}$$

with

(2) i2=j2=k2=−1,  ij=k=−ji,jk=i=−kj,  ki=j=−ik,  ijk=−1,

by Irish mathematician Sir William Rowan Hamilton in 1843, is a generalized set of complex numbers. **H**_*R*_ is an algebra over the field *R*, and this algebra is called the real quaternion algebra and the set {1, *i*, *j*, *k*} is a basis in **H**_*R*_. The elements in **H**_*R*_ take the form *a* = *a*_0_ + *a*_1_ *i* + *a*_2_ *j* + *a*_3_ *k*, where *a*_0_, *a*_1_, *a*_2_, *a*_3_ ∈ *R*, which can simply be written as *a* = *Re*⁡*a* + *Im*⁡⁡*a*, where *Re*⁡*a* = *a*_0_ and *Im*⁡⁡*a* = *a*_1_ *i* + *a*_2_ *j* + *a*_3_ *k*. The conjugate of *a* is defined as a¯=Re⁡a-Im⁡⁡a=a0-a1i-a2j-a3k, which satisfies $\bar{\bar{a}}=a,\bar{a+b}=\bar{a}+\bar{b},\bar{ab}=\bar{b}\bar{a}$ for all *a*, *b* ∈ **H**_*R*_. The norm of *a* is defined to be $\left|a\right|=\sqrt{\bar{a}a}=\sqrt{a_{0}^{2}+a_{1}^{2}+a_{2}^{2}+a_{3}^{2}}$. Some simple operation properties on quaternions are listed below:

(3) a2−2(Re⁡a)a+|a|2=0,(Im⁡⁡a)2=−Im⁡⁡a2,|ab|=|a||b|,a−1=a¯|a|2, a  is  a  nonzero  quaternion,Re⁡(ab)=Re⁡(ba).

**Theorem 1 (see [10, 15]).**

Let *a*, *b* ∈ **H**_*R*_ be quaternions. Then *a* and *b* are similar if and only if *a*_0_ = *b*_0_ and *a*_1_^2^ + *a*_2_^2^ + *a*_3_^2^ = *b*_1_^2^ + *b*_2_^2^ + *b*_3_^2^, that is, *Re*⁡(*a*) = *Re*⁡(*b*) and |*Im*⁡⁡*a*|^2^ = |*Im*⁡⁡*b*|^2^.

A complex quaternion is an element of the form *Q* = *c*_0_ *e*_0_ + *c*_1_ *e*_1_ + *c*_2_ *e*_2_ + *c*_3_ *e*_3_, where *c*_*n*_ ∈ *C*, *n* ∈ {0,1, 2,3},

(4) en2=−1, n∈{1,2,3},emen=−enem=βmnet,βmn∈{−1,1}, m≠n, m,n∈{1,2,3};

*β*_*mn*_ and *e*_*t*_ being uniquely determined by *e*_*m*_ and *e*_*n*_. We denote by **H**_*C*_ the set of the complex quaternions and **H**_*C*_ is an algebra over the field *C* and this algebra is called the complex quaternion algebra. The set {1, *e*_1_, *e*_2_, *e*_3_} is a basis in **H**_*C*_.

The element *Q* = *c*_0_ *e*_0_ + *c*_1_ *e*_1_ + *c*_2_ *e*_2_ + *c*_3_ *e*_3_ ∈ **H**_*C*_,  *c*_*m*_ ∈ *C*, *m* ∈ {0,1, 2,3}, can be written as

(5) Q=(a0+ib0)+(a1+ib1)e1+(a2+ib2)e2+(a3+ib3)e3,

where *a*_*m*_, *b*_*m*_ ∈ *R*,  *m* ∈ {0,1, 2,3}, and *i* ^2^ = −1. Therefore, we can write a complex quaternion as the form *Q* = *a* + *ib*, where *a* = *a*_0_ + *a*_1_ *e*_1_ + *a*_2_ *e*_2_ + *a*_3_ *e*_3_ and *b* = *b*_0_ + *b*_1_ *e*_1_ + *b*_2_ *e*_2_ + *b*_3_ *e*_3_ are in **H**_*R*_. The conjugate of the complex quaternion *Q* is the element $\bar{Q}=c_{0}e_{0}-c_{1}e_{1}-c_{2}e_{2}-c_{3}e_{3}$, and it satisfies

(6) $$\begin{array}{c} \bar{Q}=\bar{a}+i\bar{b}. \end{array}$$

Throughout this note, the algebra **H**_*R*_ is denoted by **H**. For the quaternion *a*, *b* ∈ **H**, if a* is defined as

(7) $$\begin{array}{c} a^{*}=a_{0}+a_{1}e_{1}-a_{2}e_{2}-a_{3}e_{3}, \end{array}$$

then it satisfies the following properties:

(8) $$\begin{array}{c} {\left(a^{*}\right)}^{*}=a,\;\;{\left(a+b\right)}^{*}=a^{*}+b^{*}. \end{array}$$

For the quaternion algebra, **H**, the map

(9) $$\begin{array}{c} \lambda :H⟶M_{4}\left(R\right),\;\;\lambda \left(a\right)=\left(\begin{array}{cccc} a_{0} & -a_{1} & -a_{2} & -a_{3}\\ a_{1} & a_{0} & -a_{3} & a_{2}\\ a_{2} & a_{3} & a_{0} & -a_{1}\\ a_{3} & -a_{2} & a_{1} & a_{0} \end{array}\right), \end{array}$$

where *a* = *a*_0_ *e*_0_ + *a*_1_ *e*_1_ + *a*_2_ *e*_2_ + *a*_3_ *e*_3_ ∈ **H**, is an isomorphism between **H** and the algebra of the matrices:

(10) $$\begin{array}{c} M_{4}\left(R\right)=\left\{\left(\begin{array}{cccc} a_{0} & -a_{1} & -a_{2} & -a_{3}\\ a_{1} & a_{0} & -a_{3} & a_{2}\\ a_{2} & a_{3} & a_{0} & -a_{1}\\ a_{3} & -a_{2} & a_{1} & a_{0} \end{array}\right),a_{0},a_{1},a_{2},a_{3}\in R\right\}. \end{array}$$

We remark that the matrix *λ*(*a*) ∈ **M**_4_(*R*) has as columns the coefficients in *R* of the basis {1, *e*_1_, *e*_2_, *e*_3_} for the elements {*a*, *ae*_1_, *ae*_2_, *ae*_3_}. The matrix *λ*(*a*) is called the left matrix representation of the element *a* ∈ **H**.

Analogously with the left matrix representation, we have for the element *a* ∈ **H** the right matrix representation:

(11) $$\begin{array}{c} \rho :H⟶M_{4}\left(R\right),\;\;\rho \left(a\right)=\left(\begin{array}{cccc} a_{0} & -a_{1} & -a_{2} & -a_{3}\\ a_{1} & a_{0} & a_{3} & -a_{2}\\ a_{2} & -a_{3} & a_{0} & -a_{1}\\ a_{3} & a_{2} & -a_{1} & a_{0} \end{array}\right), \end{array}$$

where *a* = *a*_0_ *e*_0_ + *a*_1_ *e*_1_ + *a*_2_ *e*_2_ + *a*_3_ *e*_3_ ∈ **H**.

We remark that the matrix *ρ*(*a*) ∈ **M**_4_(*R*) has as columns the coefficients in *R* of the basis {1, *e*_1_, *e*_2_, *e*_3_} for the elements {*a*, *e*_1_ *a*, *e*_2_ *a*, *e*_3_ *a*}.

**Proposition 2 (see [12]).**

For *x*, *y* ∈ **H** and *r* ∈ *R*, one has

- *λ*(*x* + *y*) = *λ*(*x*) + *λ*(*y*), *λ*(*xy*) = *λ*(*x*)*λ*(*y*), *λ*(*rx*) = *rλ*(*x*), *λ*(1) = *I*_4_,
- *ρ*(*x* + *y*) = *ρ*(*x*) + *ρ*(*y*), *ρ*(*xy*) = *ρ*(*y*)*ρ*(*x*), *ρ*(*rx*) = *rρ*(*x*), *ρ*(1) = *I*_4_,
- *λ*(*x* ^−1^) = (*λ*(*x*))^−1^, *ρ*(*x* ^−1^) = (*ρ*(*x*))^−1^, *where x* ^−1^ *is the inverse of x nonzero quaternion.*

**Proposition 3 (see [12]).**

For *x* ∈ **H**, let $\vec{x}={\left(a_{0},a_{1},a_{2},a_{3}\right)}^{t}$∈**M**_1×4_(*R*) be the vector representation of the element *x*. Therefore for all *a*, *b*, *x* ∈ **H** the following relations are fulfilled:

- $\vec{ax}=\lambda (a)\vec{x}$,
- $\vec{xb}=\rho (b)\vec{x}$,
- $\vec{axb}=\lambda (a)\rho (b)\vec{x}=\rho (b)\lambda (a)\vec{x}$,
- *ρ*(*b*)*λ*(*a*) = *λ*(*a*)*ρ*(*b*),
- det⁡(*λ*(*x*)) = det⁡(*ρ*(*x*)) = (*n*(*x*))^2^ *, where n*(*x*) = *a*_0_^2^ + *a*_1_^2^ + *a*_2_^2^ + *a*_3_^2^ *and it is the weak norm of x*.

For details about the matrix representations of the real quaternions, the reader is referred to [12].

The matrix

(12) $$\begin{array}{c} \Gamma \left(Q\right)=\left(\begin{array}{cc} \lambda \left(a\right) & -\lambda \left(b^{*}\right)\\ \lambda \left(b\right) & \lambda \left(a^{*}\right) \end{array}\right), \end{array}$$

where *Q* = *a* + *ib* is a complex quaternion, with *a* = *a*_0_ *e*_0_ + *a*_1_ *e*_1_ + *a*_2_ *e*_2_ + *a*_3_ *e*_3_ ∈ **H**,  *b* = *b*_0_ *e*_0_ + *b*_1_ *e*_1_ + *b*_2_ *e*_2_ + *b*_3_ *e*_3_ ∈ **H**, and *i* ^2^ = −1, is called the left real matrix representation for the complex quaternion *Q*. The right real matrix representation for the complex quaternion *Q* is the matrix:

(13) $$\begin{array}{c} \Theta \left(Q\right)=\left(\begin{array}{cc} \rho \left(a\right) & -\rho \left(b\right)\\ \rho \left(b^{*}\right) & \rho \left(a^{*}\right) \end{array}\right). \end{array}$$

We remark that Γ(*Q*),  Θ(*Q*) ∈ **M**_8_(*R*); see [9].

**Proposition 4 (see [9]).**

Let *a*, *x* ∈ **H** be two quaternions; then, the following relations are true.

(i) a*i = *ia, where i* ^2^ = −1.
(ii) *ai* = ia*, where i ^2^ = −1.
(iii) −a* = *i* *ai* *, where i* ^2^ = −1.
(iv) (*xa*)* = x*a*.
(v) *For X*, *A* ∈ **H**_*C*_, *X* = *x* + *iy*, *A* = *a* + *ib*,
  (14) $$\begin{array}{c} XA=xa-y^{*}b+i\left(x^{*}b+ya\right). \end{array}$$

**Proposition 5 (see [9]).**

Let *X*, *A* ∈ **H**_*C*_, *X* = *x* + *iy*, *A* = *a* + *ib* be given. Then

(15) $$\begin{array}{c} \Gamma \left(XA\right)=\Gamma \left(X\right)\Gamma \left(A\right). \end{array}$$

**Proposition 6 (see [9]).**

Let *X*, *A* ∈ **H**_*C*_, *X* = *x* + *iy*, *A* = *a* + *ib* be given. Then

(16) $$\begin{array}{c} \Theta \left(XA\right)=\Theta \left(A\right)\Theta \left(X\right). \end{array}$$

**Definition 7 (see [9]).**

Let *X* ∈ **H**_*C*_,  *X* = *x* + *iy* be given. Then

(17) $$\begin{array}{c} \vec{X}={\left(\vec{x},\vec{y}\right)}^{t}\in M_{8\times 1}\left(R\right) \end{array}$$

is the vector representation of the element *X*, where *x*, *y* ∈ **H** and $\vec{x}={\left(x_{0},x_{1},x_{2},x_{3}\right)}^{t}\in M_{4\times 1}(R)$,$\vec{y}={\left(y_{0},y_{1},y_{2},y_{3}\right)}^{t}\in M_{4\times 1}(R)$ are the vector representations of the quaternions *x* and *y*.

**Proposition 8 (see [9]).**

Let *X*, *A*, *B* ∈ **H**_*C*_, *X* = *x* + *iy*,  *x*, *y* ∈ **H** be given. Then

- $\vec{X}=\Gamma \left(X\right)\left(\begin{array}{c} 1\\ 0 \end{array}\right)$, *where *1 = *I*_4_ ∈ **M**_4_(*R*)* is the identity matrix and *0 = *O*_4_ ∈ **M**_4_(*R*)* is the zero matrix;*
- $\vec{AX}=\Gamma \left(A\right)\vec{X}$ *;*
- $\alpha \vec{y^{*}}=\vec{y}$, *where* $\alpha =\left(\begin{array}{cccc} 1 & 0 & 0 & 0\\ 0 & 1 & 0 & 0\\ 0 & 0 & -1 & 0\\ 0 & 0 & 0 & -1 \end{array}\right)\in M_{4}(R)$ *;*
- *α* ^2^ = *I*_4_ *;*
- $\vec{XA}=\left(\begin{array}{cc} 1 & 0\\ 0 & \alpha \end{array}\right)\Theta \left(A\right)\left(\begin{array}{cc} 1 & 0\\ 0 & \alpha \end{array}\right)\vec{X}$ *;*
- $\Gamma \left(A\right)\left(\begin{array}{cc} 1 & 0\\ 0 & \alpha \end{array}\right)\Theta \left(B\right)\left(\begin{array}{cc} 1 & 0\\ 0 & \alpha \end{array}\right)=\left(\begin{array}{cc} 1 & 0\\ 0 & \alpha \end{array}\right)\Theta \left(B\right)\left(\begin{array}{cc} 1 & 0\\ 0 & \alpha \end{array}\right)\Gamma \left(A\right)$ *;*
- $\vec{AXB}=\Gamma \left(A\right)\Psi \left(B\right)\vec{X}$ *, where* $\Psi \left(B\right)=\left(\begin{array}{cc} 1 & 0\\ 0 & \alpha \end{array}\right)\Theta \left(B\right)\left(\begin{array}{cc} 1 & 0\\ 0 & \alpha \end{array}\right)$.

## 3. Main Results

In this section, the complex quaternionic equations in the type

(18) $$\begin{array}{c} AX-XB=C \end{array}$$

are considered. Using the representation matrices Γ(·) and Θ(·) of complex quaternions, the necessary and sufficient conditions for the solvability and the general expression of the solutions are obtained.

According to (ii) and (v) cases in Proposition 8, (18) is equivalent to

(19) $$\begin{array}{c} \left[\Gamma \left(A\right)-\Psi \left(B\right)\right]\vec{X}=\vec{C}, \end{array}$$

where $\Psi \left(B\right)=\left(\begin{array}{cc} 1 & 0\\ 0 & \alpha \end{array}\right)\Theta \left(B\right)\left(\begin{array}{cc} 1 & 0\\ 0 & \alpha \end{array}\right)$, which is a simple system of linear equations over *R*. In order to symbolically solve it, we need to examine some operation properties on the matrix Γ(*A*) − Ψ(*B*).

**Lemma 9 .**

Let *A* = *a* + *ib*, *B* = *c* + *id* ∈ **H**_*C*_ be given, and denote *δ*(*A*, *B*) = Γ(*A*) − Ψ(*B*). Then

(i) the determinant of *δ*(*A*, *B*) is
  (20) |δ(A,B)|=([s2+(|Im⁡⁡A|−|Im⁡⁡B|)2][s2+(|Im⁡⁡A|+|Im⁡⁡B|)2])2=(s4+2s2(|Im⁡⁡A|2+|Im⁡⁡B|2)+(|Im⁡⁡A|2−|Im⁡⁡B|2)2)2,
  where *s* = *A*_0_ − *B*_0_.
(ii) if *A*_0_ ≠ *B*_0_, or |*Im*⁡⁡*A*| ≠ |*Im*⁡⁡*B*|, then *δ*(*A*, *B*) is nonsingular and its inverse can be expressed as
  (21) δ−1(A,B)=Γ−1(|B|2−|A|2+2(A0−B0)A)×(Γ(A)−Ψ(B¯));
  or
  (22) δ−1(A,B)=Ψ−1(|A|2−|B|2+2(B0−A0)B)×(Γ(A¯)−Ψ(B));
(iii) if *A*_0_ = *B*_0_ and |*Im*⁡⁡*A*| = |*Im*⁡⁡*B*|, then *δ*(*A*, *B*) is singular and has a generalized inverse as follows:
  (23) δ−(A,B)=−14|Im⁡⁡A|2δ(A,B)=−14|Im⁡⁡A|2[Γ(Im⁡⁡A)−Ψ(Im⁡⁡B)].

**Proof**

It is a known result that, for all *A*,  *B* ∈ **H**_*C*_, there are nonzero *P*, *Q* ∈ **H**_*C*_ such that

(24) A=P(A0+|Im⁡⁡A|i)P−1=PA^P−1,B=Q(B0+|Im⁡⁡B|i)Q−1=QB^Q−1,

where A^=A0+|Im⁡ A|i and B^=B0+|Im⁡⁡B|i. Now applying Propositions 5, 6, and 8 to both of them we obtain

(25) Γ(A)=Γ(P)Γ(A^)Γ(P−1),Ψ(B)=Ψ(Q−1)Ψ(B^)Ψ(Q).

Thus we can derive

(26) |δ(A,B)|=|Γ(A)−Ψ(B)|=|Γ(P)Γ(A^)Γ(P−1)−Ψ(Q−1)Ψ(B^)Ψ(Q)|=|Γ(P)||Γ(A^)−Γ(P−1)Ψ    ×(Q−1)Ψ(B^)Ψ(Q)Γ(P)|×|Γ(P−1)|=|Γ(A^)−Ψ(Q−1)Ψ(B^)Ψ(Q)|=|Ψ(Q−1)||Ψ(Q)Γ(A^)Ψ(Q−1)−Ψ(B^)||Ψ(Q)|=|Γ(A^)−Ψ(B^)|.

Consequently, substituting A^=A0+|Im⁡⁡A|i and B^=B0+|Im⁡⁡B|i into it, the proof of *i*th case in Lemma 9 is completed.

The results in Lemma 9 (ii) come from the following two equalities:

(27) [Γ(A)−Ψ(B¯)][Γ(A)−Ψ(B)] =Γ(A2)+|B|2I8−2B0Γ(A) =Γ(A2−2B0A+|B|2),[Γ(A¯)−Ψ(B)][Γ(A)−Ψ(B)] =Ψ(B2)+|A|2I8−2A0Ψ(B) =Ψ(B2+|A|2−2A0B).

Finally, under the conditions that *A*_0_ = *B*_0_ and |*Im*⁡⁡*A*| = |*Im*⁡⁡*B*|, it is easily seen that

(28) δ(A,B)=Γ(A)−Ψ(B)=Γ(Im⁡⁡A)−Ψ(Im⁡⁡B).

From it and a simple fact that (*Im*⁡⁡*A*)^2^ = (*Im*⁡⁡*B*)^2^ = −|*Im*⁡⁡*A*|^2^, we can easily deduce the following equality: *δ* ^3^(*A*, *B*) = −4|*Im*⁡ *A*|^2^ *δ*(*A*, *B*). So, the proof of *iii*th case in Lemma 9 is completed.

Based on Lemma 9, we have the following several results.

**Theorem 10 .**

Let *A* = *a* + *ib* ∈ **H**_*C*_ be given and *A* ∉ *R*. Then the general solution of the equation

(29) $$\begin{array}{c} AX=XA \end{array}$$

is

(30) X=P−1|Im⁡⁡A|2(Im⁡⁡A)P(Im⁡⁡A),

where *P* ∈ **H**_*C*_ is arbitrary.

**Proof**

According to (ii) and (v) cases in Proposition 8, (29) is equivalent to

(31) $$\begin{array}{c} \left[\Gamma \left(A\right)-\Psi \left(A\right)\right]\vec{X}=\delta \left(A,A\right)\vec{X}=0, \end{array}$$

and since |*δ*(*A*, *A*)| = 0, (31) has a nonzero solution. In that case, the general solution of (31) can be expressed as

(32) $$\begin{array}{c} \vec{X}=2\left[I_{8}-\delta^{-}\left(A,A\right)\delta \left(A,A\right)\right]\vec{P}, \end{array}$$

where $\vec{P}$ is an arbitrary vector in **H**_*C*_. Now substituting *δ* ^−^(*A*, *A*) in Lemma 9 (iii) in it, we get

(33) X→=2[I8+14|Im⁡⁡A|2δ2(A,A)]P→=2[I8+14|Im⁡⁡A|2 ×(−2[|Im⁡⁡A|2I8+Γ(Im⁡⁡A)Ψ(Im⁡⁡A)])]P→=[I8−1|Im⁡⁡A|2Γ(Im⁡⁡A)Ψ(Im⁡⁡A)]P→.

Returning it to complex quaternion form by (ii), (v), and (vii) in Proposition 8, we have (30).

**Theorem 11 .**

Let *A* = *a* + *ib*, *B* = *c* + *id* ∈ **H**_*C*_ be given. Then

(i) the linear equation
  (34) $$\begin{array}{c} AX=XB \end{array}$$
  has a nonzero solution; that is, *A* and *B* are similar, if and only if
  (35) Re⁡A=Re⁡B,  |Im⁡⁡A|=|Im⁡⁡B|;
(ii) in that case, the general solution of (34) is
  (36) X=P−1|Im⁡⁡A|2(Im⁡⁡A)P(Im⁡⁡B),
  where *P* ∈ **H**_*C*_ is arbitrary.

**Proof**

According to (ii) and (v) cases in Proposition 8, (34) is equivalent to

(37) $$\begin{array}{c} \left[\Gamma \left(A\right)-\Psi \left(B\right)\right]\vec{X}=\delta \left(A,B\right)\vec{X}=0, \end{array}$$

and this equation has a nonzero solution if and only if |*δ*(*A*, *B*)| = 0, which is equivalent, by Lemma 9 (i), to (35). In that case, the general solution of this equation can be expressed as

(38) $$\begin{array}{c} \vec{X}=2\left[I_{8}-\delta^{-}\left(A,B\right)\delta \left(A,B\right)\right]\vec{P}, \end{array}$$

where $\vec{P}$ is an arbitrary vector in **H**_*C*_. Now substituting *δ* ^−^(*A*, *A*) in Lemma 9 (iii) in it, we get

(39) X→=2[I8−δ−(A,B)δ(A,B)]P→=2[I8+14|Im⁡⁡A|2δ2(A,B)]P→=2[I8+1|Im⁡⁡A|2(−2[|Im⁡⁡A|2I8+Γ(Im⁡⁡A)Ψ(Im⁡⁡B)])]P→=[I8−1|Im⁡⁡A|2Γ(Im⁡⁡A)Ψ(Im⁡⁡B)]P→.

Returning it to complex quaternion form by (ii), (v), and (vii) in Proposition 8, we have (36).

**Theorem 12 .**

Let *A* = *a* + *ib*, *B* = *c* + *id* ∈ **H**_*C*_,  *a*, *b*, *c*, *d* ∈ **H**, be given with *A* and *B*  being not similar; that is, *Re*⁡*A* ≠ *Re*⁡*B* or |*Im*⁡⁡*A* | ≠ |*Im*⁡⁡*B*|. Then (18) has a unique solution

(40) X=(2(A0−B0)A−|A|2+|B|2)−1(AC−CB¯)=(A¯C−CB)(2(B0−A0)B+|A|2−|B|2)−1.

**Proof**

Under the assumption of this theorem, *δ*(*A*, *B*) = Γ(*A*) − Ψ(*B*) is nonsingular by Lemma 9 (ii). Hence (19) has a unique solution as follows:

(41) X→=δ−1(A,B)C→=Γ−1(2(A0−B0)A−|A|2+|B|2)×(Γ(A)−Ψ(B¯))C→X→=δ−1(A,B)C→=Γ−1(2(A0−B0)A−|A|2+|B|2)×(Γ(A¯)−Ψ(B))C→,

and the result follows using (ii) and (v) of Proposition 8.

**Theorem 13 .**

Let *A* = *a* + *ib* ∈ **H**_*C*_ be given. Then the general solution of the equation

(42) $$\begin{array}{c} AX-XA=C \end{array}$$

is

(43) X=14|Im⁡⁡A|2(CA−AC)+P−1|Im⁡⁡A|2(Im⁡⁡A)P(Im⁡⁡A),

where *P* ∈ **H**_*C*_ is arbitrary.

**Proof**

According to (ii) and (v) cases in Proposition 8, (42) is equivalent to

(44) $$\begin{array}{c} \left[\Gamma \left(A\right)-\Psi \left(A\right)\right]\vec{X}=\delta \left(A,A\right)\vec{X}=\vec{C}, \end{array}$$

and since |*δ*(*A*, *A*)| = 0, (44) has a nonzero solution. In that case, the general solution of (44) can be expressed as

(45) $$\begin{array}{c} \vec{X}=\delta^{-}\left(A,A\right)\vec{C}+2\left[I_{8}-\delta^{-}\left(A,A\right)\delta \left(A,A\right)\right]\vec{P}, \end{array}$$

where $\vec{P}$ is an arbitrary vector in **H**_*C*_. Now substituting *δ* ^−^(*A*, *A*) in Lemma 9 (iii) in it, we get

(46) X→=δ−(A,A)C→+2[I8−δ−(A,A)δ(A,A)]P→=−14|Im⁡⁡A|2δ(A,A)C→+2[I8−δ−(A,A)δ(A,A)]P→=−14|Im⁡⁡A|2(Γ(A)−Ψ(A))C→+[I8−1|Im⁡⁡A|2Γ(Im⁡⁡A)Ψ(Im⁡⁡A)]P→.

Returning it to complex quaternion form by (ii), (v), and (vii), we have (43).

**Theorem 14 .**

Let *A* = *a* + *ib*, *B* = *c* + *id* ∈ **H**_*C*_,  *a*, *b*, *c*, *d* ∈ **H**, be given with *A* ~ *B*. Then (18) has a solution if and only if

(47) $$\begin{array}{c} AC=C\bar{B}, \end{array}$$

in which case the general solution of (18) can be written as

(48) X=14|Im⁡⁡A|2(CB−AC)+P−1|Im⁡⁡A|2(Im⁡⁡A)P(Im⁡⁡B),

where *P* ∈ **H**_*C*_ is arbitrary.

**Proof**

According to (ii) and (v) cases in Proposition 8, (18) is equivalent to

(49) $$\begin{array}{c} \left[\Gamma \left(A\right)-\Psi \left(B\right)\right]\vec{X}=\delta \left(A,B\right)\vec{X}=\vec{C}. \end{array}$$

This equation is solvable if and only if

(50) $$\begin{array}{c} \delta \left(A,B\right)\delta^{-}\left(A,B\right)\vec{C}=\vec{C}, \end{array}$$

which is equivalent to

(51) Γ(Im⁡⁡A)Ψ(Im⁡⁡B)C→=|Im⁡⁡A|2C→.

Returning it to complex quaternion form by (ii) and (v) cases in Proposition 8, we obtain

(52) (Im⁡⁡A)C(Im⁡⁡B)=|Im⁡⁡A|2C,

which is equivalent to *C*(*Im*⁡⁡*B*) = −(*Im*⁡⁡*A*)*C*, and then (47). In that case the general solution of (49) can be expressed as

(53) $$\begin{array}{c} \vec{X}=\delta^{-}\left(A,B\right)\vec{C}+2\left[I_{8}-\delta^{-}\left(A,B\right)\delta \left(A,B\right)\right]\vec{P}, \end{array}$$

where $\vec{P}$ is an arbitrary vector in **H**_*C*_. Returning it to complex quaternion form, we find (48).

## 4. Conclusions

Starting from known results and referring to the real matrix representations of the complex quaternions, in this paper we have investigated solutions of some linear equations with two terms and one unknown by the method of matrix representations of complex quaternions over the complex quaternion field.

The methods and results developed in this paper can also extend to complex octonionic equations. We will present them in another paper.
